# Three's Company: Coexistence of Silicosis, Scleroderma, and Sjogren Syndrome in a Single Patient

**DOI:** 10.1155/2022/4487638

**Published:** 2022-05-27

**Authors:** Alexis Ziebelman, Shir Raibman-Spector, Omer Gendelman

**Affiliations:** ^1^Department of Medicine “B”, Sheba Medical Center, Tel-Hashomer, Ramat Gan, Israel; ^2^Sackler Faculty of Medicine, Tel Aviv University, Tel-Aviv, Israel; ^3^Department of Medicine “E”, Sheba Medical Center, Tel-Hashomer, Ramat Gan, Israel; ^4^Rheumatology Unit, Sheba Medical Center, Tel-Hashomer, Ramat Gan, Israel

## Abstract

We describe a patient who presented with silicosis, scleroderma, and Sjogren syndrome all at the same time. The diagnoses in this case are all associated with continuous exposure to crystalline silica at the patient's workplace. The following report discusses this unique presentation.

## 1. Introduction

The etiology of autoimmune diseases is complex and multifactorial [1]. Genetics has been proven to play a role in the development of autoimmune diseases; however, it is not the sole contributing risk factor. Over the past few years, environmental factors have been found to play a complementary and significant role in the initiation and propagation of different autoimmune diseases and can be linked to up to 70% of all autoimmune diseases [2].

Environmental exposure to crystalline silica is a well-known occupational hazard among those involved in stone quarrying, mining, and sand blasting, and continuous exposure is associated with an increased risk for a variety of silica-related diseases [1, 3]. Silicosis is an inflammatory lung disease that results from inhalation and retention of crystalline silica in the lung [4]. Several forms of clinical silicosis have been reported:

1. Acute silicosis occurring after relatively short (a few months to as long as 2 years) but intense exposure to high concentrations of silica. Clinical symptoms include fatigue, cough, dyspnea, pleuritic pain, and uncommonly, rapid progression occurs, resulting in respiratory failure [5].
2. Accelerated silicosis occurs within 10 years from the initial exposure to a large amount of respirable crystalline. Clinical manifestations are variable, ranging from individuals with only abnormal chest imaging without symptoms to those presenting with cough and dyspnea [6].
3. Chronic silicosis, subdivided into simple (nodular) silicosis and progressive massive fibrosis, occurs after 15–20 years of moderate to low exposure. Clinical symptoms may not appear until years after exposure, ranging from mild cough to severe dyspnea and respiratory failure [7].

In addition, exposure to inhaled silica possesses risks for other pulmonary diseases including lung cancer and tuberculosis [8].

Exposure to crystalline silica has also been found to be associated with the development of various autoimmune diseases including systemic lupus erythematosus (SLE), rheumatoid arthritis, systemic sclerosis, and antineutrophil cytoplasmic antibody (ANCA)-related vasculitis [9].

Autoimmune/inflammatory syndrome induced by adjuvants (ASIA) encompasses diverse inflammatory phenomena that occur due to exposure to an adjuvant. The disease spectrum can vary widely in clinical presentation and extent of severity of the manifestations. It has been reported that some individuals develop constitutional symptoms, antibody production, or worsening of current inflammatory conditions in response to exposure to and adjuvant. Watad et al. first introduced this syndrome in 2011 and considered silica to be an adjuvant [10]. There are also several case reports of women presenting with autoimmune symptoms after silicone breast implantation. De Carvalho and Shoenfeld described a case report of the first patient with silicone breast implantation to develop systemic sclerosis sine scleroderma (ssSSc), an atypical form of systemic sclerosis that lacks skin involvement [11]. Yet, in case-control studies, there is no association seen between the presence of connective tissue disorders and silica exposure [12].

In the current case report, we describe a patient who simultaneously developed three different inflammatory and autoimmune diseases after prolonged exposure to crystalline silica.

The patient is a 52-year-old male, who worked as a marble mason for over 20 years. His medical history is significant for type II diabetes mellitus and smoking. He presented with a 7-month history of polyarthralgia and numbness, mainly involving the palms and feet and accompanied by morning stiffness lasting for one hour. He also suffered from episodic bluish discoloration of fingers and toes upon exposure to cold, which was suggestive of Raynaud's phenomenon. In addition, he noticed recurrent small sores on the tip of his fingers. Finally, he also complained of dry mouth and mild dyspnea over the last year. The patient was then hospitalized for further work-up.

**Figure 1:**
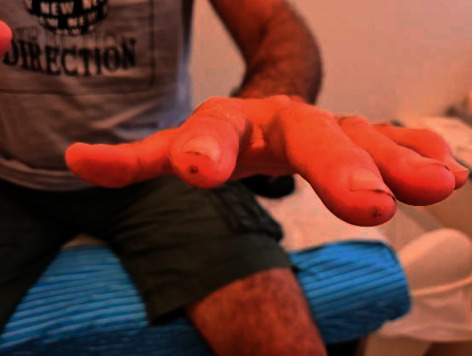
Pitting scars at the tip of the digits.

**Figure 2:**
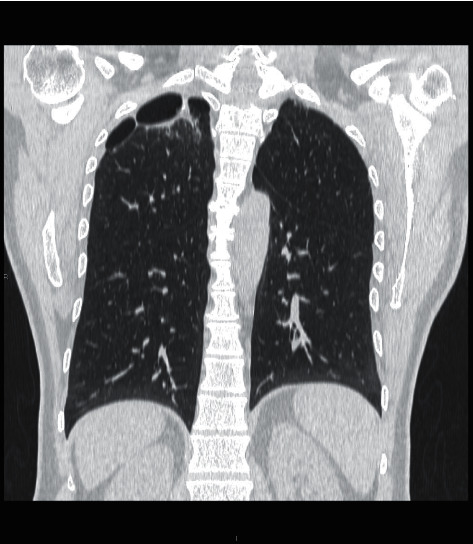
Chest CT demonstrating apical fibrosis.

Upon admission, examination of the head and neck revealed an enlarged, smooth, nontender left parotid gland. Physical examination of the hand joints revealed puffy fingers and arthralgia without signs of arthritis over the MCP and PIP joints. Sclerodactyly distal to the MCPs was noted bilaterally, accompanied by pitting scars at the tip of the digits. Pigmentary changes with areas of hypopigmentation were observed over the MCP and PIP joints (Figures 1 and 2). Additionally, the perioral skin was tight with reduced oral aperture.

Laboratory investigations including complete blood count, urinalysis, liver, and renal function tests were within normal limits. The erythrocyte sedimentation rate (ESR) was elevated: 85 mm/h. Rheumatoid factor (RF) and anticyclic citrullinated peptide (CCP) were negative. Antinuclear antibodies (ANA), homogeneous and speckled, were positive with the titer of 1 : 1280 together with high titers of both anti-Ro/SSA and anti-La/SSB (>8, normal 0–0.99) and Scl-70 (>8, normal 0–0.99). HCV Ab and HBV surface Ag and core-Ab were negative.

**Figure 3:**
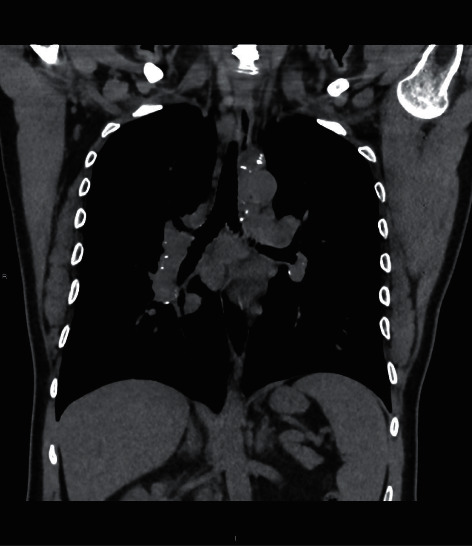
Chest CT: mediastinal and hilar adenopathy with coarse eggshell classification.

Chest X-ray demonstrated a bulla and small rounded consolidation in the right upper lobe, while high resolution computed tomography (HRCT) of the chest revealed bilateral apical fibrosis, upwards hilar traction, mediastinal and hilar adenopathy with coarse eggshell calcification, and esophageal dilation (Figure 3).

Spirometry and lung function were as follows: FEV1 77%, FVC 70%, DLCO 84%, TLC 93%, FRC 100%, RV 131%, and SaO_2_ 98% on room air reflecting a restrictive lung disease pattern.

Accordingly, the patient was diagnosed with pulmonary silicosis, scleroderma, and Sjogren syndrome.

The patient was prescribed methotrexate (10 mg/week), prednisone (5 mg/day), and amlodipine (10 mg/day) and discharged for ambulatory follow-up in the rheumatology and pulmonology outpatient clinics.

## 2. Discussion

The diagnoses of pulmonary silicosis, scleroderma, and Sjogren syndrome observed in this case are all associated with continuous exposure to crystalline silica at the patient's workplace.

The connection between silicosis and inhalation of crystalline silica is well established [3]. The silica particles are phagocyted by macrophages, which in turn stimulates the production of proinflammatory cytokines such as IL-1 and TNF. In addition, activation of the NALP3 protein inflammasome induces regulatory T cells to express cytotoxic T-lymphocyte antigen 4, IL-10, and transforming growth factor-beta (TGF-*β*). This process results in lung tissue inflammation and fibrosis [13].

The development of systemic sclerosis after silica exposure is a newer concept, initially described by Erasmus et al. in 1957 based on Witwatersrand gold miners who developed scleroderma [14]. Today, silica exposure is considered a major environmental risk factor for scleroderma [15,16]. A case–control study including 80 cases and 160 matched controls investigating the relation between systemic sclerosis and occupational exposure found a significantly increased OR (odds ratio) for systemic sclerosis following exposure to crystalline silica (OR 5.57, 95% CI 1.69–18.37) [17]. In a meta-analysis by Memuric et al. encompassing 16 series (1101 cases in 3 cohorts and 9 case–control series), assessing the association between silica exposure and scleroderma, only three studies reported lower risk or no association between silica exposure and scleroderma. The combined estimator of relative risk (CERR) for scleroderma was 3.20 (95% CI 1.89–5.43), reaching 15.49 (95% CI 4.54–52.87) for the cohort studies included in the analysis [16]. Finally, a prospective study evaluating the association between systemic sclerosis and occupational exposure to various chemicals and pollutants found an increased risk (OR 5.32, *p* < 0.0001) for systemic sclerosis following exposure to crystalline silica [18].

The direct mechanism of how crystalline silica induces scleroderma is unclear. However, there is a theory that silica particles stimulate and induce responder T cells, but not T regulatory cells, to avoid apoptosis. This imbalance shifts the immune system towards autoimmunity, contributing to the development of scleroderma [19]. Interestingly, scleroderma associated with silica exposure differs clinically from “idiopathic” scleroderma. According to a systematic review by Freire et al., scleroderma associated with silica exhibits a male predominance, is more of the diffuse subtype, and shows lower survival [20]. In a retrospective study of clinical and laboratory features of patients with scleroderma associated with silica (“Erasmus syndrome”), the majority of patients were male (78%), smokers (68%), and presented with diffuse scleroderma (67%). Antinuclear antibodies were positive in all patients, while antitopoisomerase I was positive in 44% [21]. It is critical to mention that the observed male predominance might be a reflection of the higher rate of man workers in the mining and dusty industries [22].

Compared with scleroderma, the causality between Sjogren syndrome and silicone exposure is less well understood. There are a few case reports that reported an association, yet the underlying mechanism remains unclear [23, 24]. Colafrancesco et al. considered silicone as an adjuvant, a substance that acts to accelerate, prolong, or enhance antigen-specific immune responses and capable to induce the ASIA syndrome (an autoimmune/inflammatory syndrome induced by adjuvants). The ability of adjuvants to initiate an immune response in the glandular epithelium and the shared symptoms in both Sjogren's and ASIA syndromes may explain the impact of silicone on the pathogenesis of Sjogren syndrome [25].

It is also critical to note that in patients with scleroderma, an association exists with Sjogren syndrome that can reach up to 10%, irrespective of occupational exposure [26]. Furthermore, anti-Ro antibody positive disease, such as in this patient, has been noted to account for 20% of scleroderma cases [27]. Anti-Ro positivity is itself a worse prognostic factor and can be viewed independently of the silica stimulation pathway and must be considered in this patient [28].

Exposure to crystalline silica is a significant occupational hazard for a variety of immune-mediated diseases. Scleroderma and Sjogren syndrome, appearing simultaneously in our patient after continuous exposure to silica, are both noncurable autoimmune diseases, associated with significant morbidity and increased mortality. As modifiable risk factors for autoimmune diseases are scarce, active surveillance, screening, and protective measures must be initiated in those with hazardous exposure to crystalline silica. Hopefully, this will decrease the prevalence of the diseases associated with such exposure in the future.
